# Obesity and Skin Carotenoid Score in Children from Center–Southern Italian Regions

**DOI:** 10.3390/antiox14040448

**Published:** 2025-04-08

**Authors:** Giuseppina Augimeri, Giovanna Caparello, Fabrizio Ceraudo, Francesca Meringolo, Ludovica Mazzitelli, Maria Vittoria Iovino, Giuseppe Morino, Daniela Bonofiglio

**Affiliations:** 1 Department of Pharmacy, Health and Nutritional Sciences, University of Calabria, Arcavacata di Rende, 87036 Cosenza, Italy; giuseppina.augimeri@unical.it (G.A.); caparello.giovanna@gmail.com (G.C.); fabrizio.cer96@gmail.com (F.C.); francesca.meringolo@outlook.it (F.M.); 2 Fondazione Pancrazio, Via Riccardo Misasi, 86, 87100 Cosenza, Italy; mazzitelli@fondazionepancrazio.it (L.M.); iovino@fondazionepancrazio.it (M.V.I.); 3 Nutrition Unit, Bambino Gesù, Children’s Hospital IRCCS, 00165 Rome, Italy; gsmorino53@gmail.com; 4 Centro Sanitario, University of Calabria, Arcavacata di Rende, 87036 Cosenza, Italy

**Keywords:** childhood obesity, Mediterranean Diet, skin carotenoid levels, Veggie Meter^®^, natural antioxidants

## Abstract

Childhood obesity represents a public concern worldwide. Evidence indicates that fruits and vegetables (FV) consumed as part of the daily diet reduce the global burden of obesity. Indeed, FV are rich in bioactive compounds, including carotenoids, which exert health benefits as very potent natural antioxidants. Here, we compared the anthropometric characteristics and the skin carotenoid content between two schoolchildren populations from Southern and Central Italy to evaluate their dietary habits. A sample of 121 and 124 schoolchildren from primary schools in Central and Southern Italy, respectively, was recruited. All participants underwent anthropometric measurements and assessment of the adherence to the Mediterranean Diet (MD) by the KIDMED questionnaire and the skin carotenoid score by Veggie Meter^®^. The mean body mass index (BMI) was significantly lower in participants from Central Italy than in those from Southern Italy. A significantly higher percentage of overweight and obesity was found in the overall sample from Southern than in Central Italy. The adherence to the MD was in the average range for both populations, with no gender-related differences. The carotenoid score was higher and negatively correlated with BMI in the schoolchildren from Central Italy. In multiple regression analyses, skin carotenoids were positively associated with the consumption of fruit in the entire sample. This study suggests the importance of increasing FV intake, particularly in Southern Italy, as a strategy for preventingof obesity during the whole lifespan. Further studies are essential to better understand the influence of skin carotenoids on different variables and their potential role as indicators of health status in children.

## 1. Introduction

Since 2007, the World Health Organization’s (WHO) Childhood Obesity Surveillance Initiative (COSI) has been monitoring overweight and obesity among European school-age children with the cut-offs of the International Obesity Task Force (IOTF) as standardized measurements of weight and height. In 2018–2020, COSI data collection involved 33 countries, among which Italy had the highest percentages of overweight children together with other countries in the Mediterranean area [1]. As an integral part of the COSI, the Italian Surveillance System called “OKKIO alla SALUTE” has reported that the percentage of overweight (not including obesity) children was 19.0%, while obese children were 9.8%, including 2.6% with severe obesity, in 2023 [2]. Collectively, obesity prevalence was higher in Southern than in Centre–North Italy in both genders. Accordingly, skipping breakfasts, drinking sugar-sweetened beverages, and reducing the daily intake of fruits and vegetables (FV) displayed a similar geographical trend in Italy [2].

A large body of evidence indicates that unhealthy eating behaviors, usually formed in early childhood, are crucial factors for the development of obesity among children [3,4,5]. More specifically, FV intake is a key modifiable factor for the prevention of obesity and a plethora of metabolic and chronic diseases, also known as non-communicable diseases (NCDs) [6,7,8,9,10]. As such, to reduce the global burden of NCDs, the WHO recommended consuming more than 400 g of FV per day [11] because of their high concentrations of vitamins and minerals, dietary fibers, and a wide range of beneficial non-nutrient substances including plant sterols, flavonoids, and antioxidants. Thus, consuming a variety of FV helps to ensure an adequate intake of many of these essential nutrients with antioxidant properties, including carotenoids [12].

The most common forms of carotenoids found in the diet, representing over 95% of the total blood carotenoids in human serum, include β-carotene, α-carotene, β-cryptoxanthin, lutein, zeaxanthin, and lycopene, of which the first three are provitamin A carotenoids. β-carotene is mainly present in orange, yellow, and green leafy vegetables, such as carrots, spinach, and lettuce. Lycopene is abundant in red FV, including tomato and watermelon. Lutein has been found in green leafy vegetables, such as broccoli and parsley. Consumption of carotenoids has been linked to a reduced risk of several chronic diseases, including cardiovascular diseases, type 2 diabetes mellitus, and different types of cancer [13,14]. One of the most important properties of carotenoids is their scavenging activity against singlet oxygen (^1^O_2_) and other reactive oxygen species (ROS), including peroxyl radicals. Carotenoids quench ^1^O_2_ mainly through a physical mechanism in which the energy is transferred between the molecules. This ability depends on the number of conjugated double bonds present in the carotenoid chemical structure. Lycopene is considered the most efficient quencher of ^1^O_2_ in humans, accounting for up to 30% of total carotenoids [15]. Peroxyl radicals are produced during lipid peroxidation and induce cell membrane damage and death. Carotenoids, as scavenging molecules, limit the propagation reaction, deactivating the peroxyl radicals [16]. Several factors, from the food matrix and cooking methods to polymorphisms in the genes coding for the proteins that regulate intestinal absorption, result in individual variability in the metabolism and transport of carotenoids and vitamin A [17]. Generally, carotenoids are absorbed by enterocytes either by passive diffusion or receptor-assisted transport and are incorporated into chylomicrons for secretion in the lymphatic system. The distribution of carotenoids in the human body varies depending on the specific carotenoids, with a common tendency for accumulation in the liver, skin, adrenal glands, and, particularly, adipose tissue. Carotenoids are detectable in human fluids, commonly in serum, and in different tissues where they create a network strengthening the effectiveness of antioxidant defense [18,19]. Of note, serum carotenoid measurements are indicative of short-term dietary intakes of antioxidants, while the accumulations in body tissues, such as in the skin, reaching a steady state indicate a long-term strength of the antioxidant system. Indeed, skin carotenoids offer an alternative measurement for the consumption of FV, allowing the validation of the most traditional dietary assessment methods.

An effective method is the spectroscopy-based measurement using the Veggie Meter^®^, which represents a simple, non-invasive objective tool for determining skin carotenoid concentrations. The aforementioned advantages of the Veggie Meter^®^ support the use of this device in the research setting to assess public health and nutrition education interventions [20]. In this context, we have recently published results from the “Dammi il 5” project conducted in the schoolchildren population of Southern Italy, highlighting the positive influence of daily FV intake and school lunch attendance on skin carotenoids measured by Veggie Meter^®^ [21]. Here, we compared anthropometric characteristics and skin carotenoid scores between two schoolchildren populations from Calabria and Lazio regions, located in Southern and Central Italy, respectively, to evaluate their dietary habits. Moreover, by creating multiple regression models, we evaluated variables that affect skin carotenoids in schoolchildren, shedding light on the beneficial effects of natural antioxidants on human health.

## 2. Materials and Methods

### 2.1. Study Design

As part of the scientific project “Dammi il 5”, 121 (55 boys and 66 girls) and 124 subjects (58 boys and 66 girls) aged between 8 and 10 years old were recruited at the primary school “Crispi” of Rome, Lazio Region, and “L. Plastina Pizzuti” of Cosenza, Calabria Region, Italy. Exclusion criteria from the study were metabolic and chronic diseases, liver diseases, any kind of cognitive or physical/motor limitation, and any kind of restrictive diet (i.e., hypocaloric, low carbohydrate, and low fat). The study’s objectives were presented to all participants’ parents. Written informed consent was obtained from parent’s participants before the enrollment of children in this study. Participants underwent anthropometric and skin carotenoid measurements. Moreover, schoolchildren participated with the help of their parents in scientific research activities to complete the KIDMED questionnaires to assess Mediterranean Diet adherence, as described previously [21]. The data were collected from March 2024 to April 2024. The rationale of the research project and the adequacy of the protocol, according to the guidelines laid down in the Declaration of Helsinki, were approved by the Ethics Committee of the University of Calabria, Italy (#53519/2022).

### 2.2. Anthropometric Parameters

Nutritionists gathered anthropometric data, including weight and height, using established and standardized procedures [22]. Particularly, weights were determined using the TANITA BC-545 N, whereas height was determined using a Seca stadiometer (Model 206, Seca Deutschland, Hamburg, Germany), as previously described [21]. The BMI was calculated by dividing the body weight expressed in kilograms by the square of the height in meters [BMI = weight (kg)/height^2^ (m)]. The standard cut points were used to categorize the population into three BMI categories based on gender and age. In particular, children from Lazio were stratified based on the cut-off for 7.5 years old children: underweight–normal weight: <18.16 for boys, 18.03 for girls; overweight: 21.09 for boys, 21.01 for girls; obese: >21.10 for boys and >21.02 for girls. Children from Calabria were stratified based on the cut-off for 8.5 years old children: underweight–normal weight: <18.76 for boys, 18.69 for girls; overweight: 22.17 for boys, 22.18 for girls; obese: >22.18 for boys and >22.19 for girls. The BMI Z-score was obtained using the following formula: [BMI Z-score = [(BMI/M(t))L(t) − 1]/L(t)S(t)].

### 2.3. Adherence to the Mediterranean Diet Using the KIDMED Questionnaire

Using the validated Mediterranean Diet Quality Index (KIDMED) test, we assessed adherence to the Mediterranean Diet. The KIDMED questionnaire consists of 16 items: 4 questions indicating unhealthy dietary habits, each assigned a score of −1, and 12 questions reflecting positive eating behaviors, each given a score of +1. The total KIDMED score ranges from <0 to ≤12, with higher values signifying greater adherence to the Mediterranean Diet. Based on the KIDMED score, we categorized our study population into three groups: high adherence (≥8 points), moderate adherence (4 to 7 points), and low adherence (≤3 points) [23].

### 2.4. Measurement of Skin Carotenoid Content

Skin carotenoid levels were measured using the Veggie Meter^®^ (Longevity Link Corporation, Salt Lake City, UT, USA), a spectroscopy-based method, according to the manufacturer’s instructions [20]. Briefly, the device’s calibration was performed with the provided dark and white reference materials before the data collection. Then, the skin carotenoid measurement was executed on the index finger of the non-dominant hand of each subject, after hand washing, in a single measurement mode. The subjects inserted their finger into the device applying a modest pressure to decrease the blood perfusion of the measured tissue volume, which might interfere with the measurement of skin carotenoid content. A computer analyzed the light that was reflected from the finger and provided a score on a spectral range from 0 to 800, with higher scores indicating higher skin carotenoid scores. Values under 100 were excluded, while values around 400 were considered optimal.

### 2.5. Statistical Analysis

Data were reported as mean and standard deviation (SD) for quantitative variables, while categorical variables were reported with absolute frequency and percentage. The carotenoid score was also reported as the median with 25% and 75% percentile values. Statistical differences and correlations with BMI were evaluated using parametric tests (Student’s *t*-test and Chi-square test) and Pearson’s linear correlation index in the GraphPad-Prism 7.00 software program, respectively. Multivariate linear regression models were used to test the association between carotenoid score, as an independent variable, and different parameters, including gender, BMI, diet, and place of living as dependent variables, in IBM SPSS v. 25. Sample size was calculated using a 95% confidence interval (CI) and a 5% error. A minimum number of 120 participants from primary schools was requested. Statistical significance was set at *p* < 0.05.

## 3. Results

### 3.1. Sample Characteristics

The main characteristics of the total sample population and categorized by gender are reported in Table 1. A total of 121 and 124 schoolchildren were enrolled in the primary schools in the Lazio and Calabria regions, respectively. The mean BMI and BMI-Z scores were significantly higher in children from Calabria than in those from Lazio both in the total population (BMI, *p* < 0.0001, BMI-Z score, *p* < 0.0001) and categorized by gender (*p* < 0.0001) (Table 1).

As expected, a significantly higher percentage of overweight and obese participants were found in the population from Calabria than in Lazio (BMI: *p* < 0.0001; BMI-z score *p* < 0.0001). Similar results were obtained by stratifying the two sample populations based on gender according to BMI and BMI-Z scores (Table 2).

### 3.2. Adherence to the Mediterranean Diet in the Sample Population

As shown in Table 3, an average adherence to the Mediterranean Diet was found in both schoolchildren from Lazio and Calabria, with no differences between the two sample populations. Moreover, no gender-related differences were observed in the mean of the KIDMED score (Table 3).

**Figure 1:**
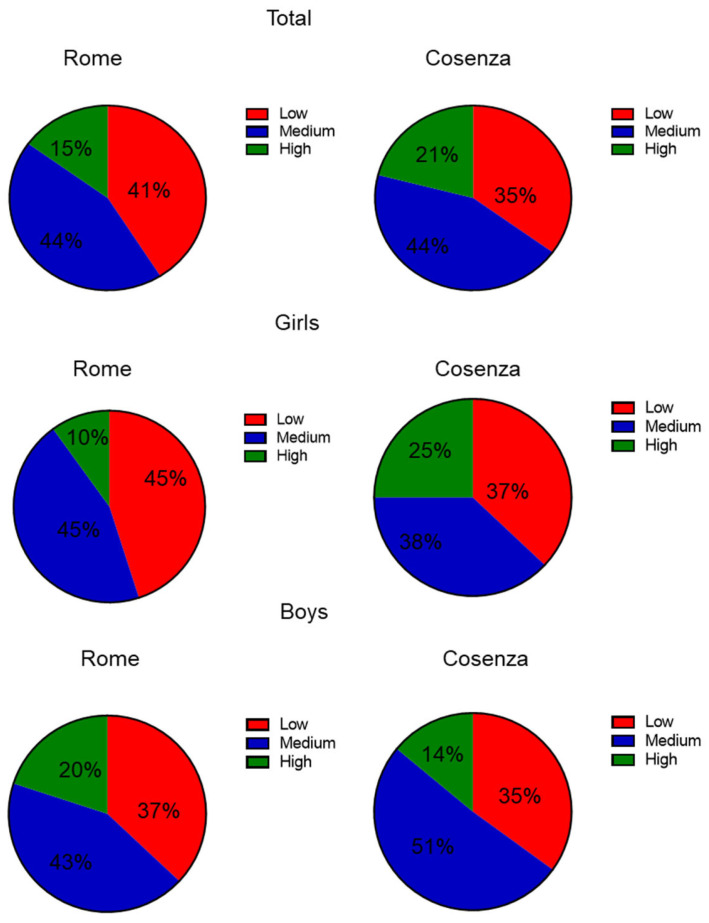
Stratification of the total sample divided by gender with respect to the Mediterranean Diet adherence evaluated by the KIDMED test. Based on the KIDMED score, we categorized our study population into three groups: high adherence (≥8 points), moderate adherence (4 to 7 points), and low adherence (≤3 points).

By stratifying the population based on the KIDMED score, we observed that 41%, 44%, and 15% of participants had low, medium, and high adherence to the Mediterranean Diet, respectively, in the schoolchildren from Lazio. Similarly, among children from Calabria, 35% had low adherence, 44% had medium adherence, and 21% had high adherence. A similar trend was recorded when categorizing the population by gender in both regions (Figure 1).

However, several differences were found by estimating the compliance rates for each Mediterranean Diet recommendation reported in the KIDMED questionnaire in the total children population for either unhealthy dietary habits or positive eating behaviors (Table 4). For unhealthy dietary habits, a significantly lower percentage of participants from Lazio declared to “go to a fast-food more than once per week” (*p* < 0.0001) and “take sweets and candy several times every day” (*p* < 0.0006). On the contrary, for positive eating behaviors, a higher percentage of schoolchildren from Lazio have “whole cereals or whole-grains for breakfast” (*p* < 0.0001), and “consumes whole-grain pasta or whole-grain rice almost every day” (*p* < 0.0001). Regarding the items on the FV intake, a significantly higher percentage of schoolchildren reported to “take a fruit every day” in the population from Lazio (*p* = 0.047).

### 3.3. Carotenoid Score in the Sample Population

**Figure 2:**
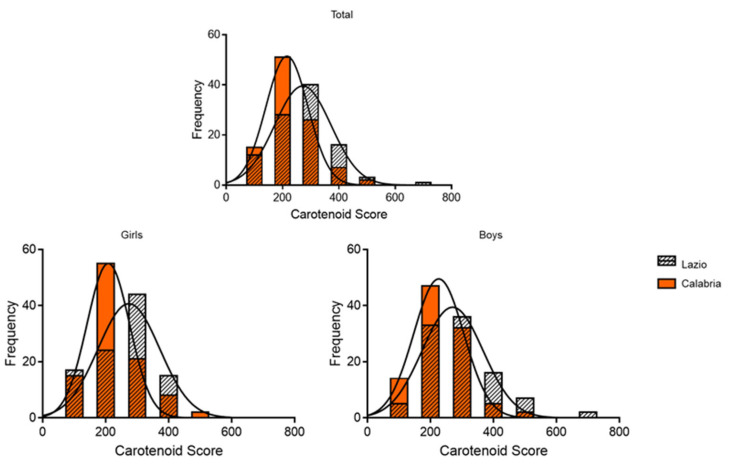
Distribution of the carotenoid scores with Veggie Meter^®^ in the total population and categorized by gender. The skin carotenoid scores ranged from 256 to 384 (median, 310) in the total schoolchildren population from Lazio, while ranging from 218 to 322 (median, 263) in Calabria. In girls, the skin carotenoid scores ranged from 240.8 to 349.5 (median, 309) for Lazio and ranged from 213.5 to 314.5 (median, 257.5) for Calabria. In boys, it ranged from 269 to 403 (median, 316) for Lazio and ranged from 221 to 323.5 (median, 275) for Calabria.

The distribution of carotenoid scores, measured by Veggie Meter^®^, was reported in a histogram that represents both the total populations and categorized by gender (Figure 2). The skin carotenoid scores ranged from 256 to 384 (median, 310) in the total schoolchildren population from Lazio, while ranging from 218 to 322 (median, 263) in Calabria. In girls, the skin carotenoid scores ranged from 240.8 to 349.5 (median, 309) for Lazio and ranged from 213.5 to 314.5 (median, 257.5) for Calabria. In boys, it ranged from 269 to 403 (median, 316) for Lazio and ranged from 221 to 323.5 (median, 275) for Calabria. It is noteworthy that the mean carotenoid score was significantly higher in the entire sample of schoolchildren from Lazio than in Calabria (*p* = 0.0005) (Figure 2 and Table 5).

Categorizing the population by gender, a significantly higher carotenoid score was found in boys from Lazio than in Calabria (*p* = 0.001), whereas no differences were found between Calabria and Lazio girls. Moreover, a gender-related difference was found in the schoolchildren from Lazio, with boys showing significantly higher carotenoid scores than girls (*p* = 0.03) (Table 5). Moreover, when considering the items from the KIDMED questionnaire “do you consume more than 2 fruits/day” (item #1) and “do you consume more than 2 servings of vegetables/day” (item #2), we found a significantly higher carotenoid score in children consuming more than two fruits/day in both regions, whereas no significant differences were found in the carotenoid score according to vegetable intake frequency (Table 6).

### 3.4. Correlation Between Carotenoid Score and Different Parameters

**Figure 3:**
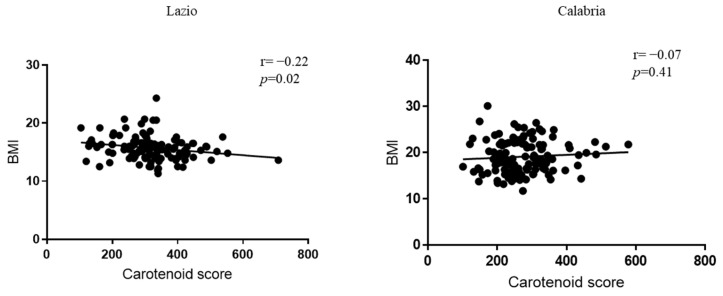
Correlation between BMI and carotenoid score in the schoolchildren from Lazio and Calabria. The correlation between skin carotenoid content and BMI was analyzed using Pearson’s correlation test. For each linear regression graph, the correlation coefficient (r) and the statistical significance (*p)* are reported.

To test whether the skin carotenoid score might have an association with different parameters, we first estimated the correlations between the carotenoid score and BMI by Pearson’s correlation in both populations. We observed that skin carotenoids were negatively correlated with the BMI in the schoolchildren from Lazio (r = −0.22, *p* = 0.02), whereas no significant correlation was observed between these two parameters in the participants from Calabria (Figure 3).

Moreover, we performed multiple linear regression analysis using the carotenoid score as the independent variable, and gender, BMI, the consumption of FV, and the region of residence as dependent variables. We found that the carotenoid score was positively associated with the consumption of fruit (*p* = 0.003) (Table 7).

## 4. Discussion

In this study, we compared anthropometric characteristics and skin carotenoid scores between two primary schoolchildren populations from Calabria and Lazio regions, located in Southern and Central Italy, respectively. We found a significantly higher percentage of overweight and obesity in the overall sample from Southern Italy than in Central Italy, while the carotenoid score was significantly higher and negatively correlated with BMI in the schoolchildren from Central Italy. In multiple regression analyses, skin carotenoids were positively associated with the consumption of fruit, as a natural source of dietary antioxidants, in the entire population sample.

Mounting evidence supports the beneficial effects of a healthy diet to prevent childhood obesity and the development of obesity-related diseases in adulthood [24,25]. A healthy diet, such as the Mediterranean Diet, includes an adequate intake of whole grains, milk and dairy products, fish and poultry, and an abundant amount of FV, whereas a dietary pattern rich in saturated fats, sugars, and processed meats is recognized as being unhealthy and obesogenic [26,27]. In both population samples, we found an average adherence to the Mediterranean Diet, regardless of gender, as previously reported in children and adolescents [22,28,29] as well as in adults [30,31,32] in the same geographical area. However, the anthropometric characteristics of the two populations were significantly different. In particular, agreeing with the data recently reported by the National Surveillance System called “OKKIO alla Salute” [2,33], we found a significantly higher percentage of overweight and obesity in participants from Calabria than in Lazio. This conflicting result might be explained by considering that even if the adherence to the Mediterranean Diet is the same in the two regions, the portion size of food in Calabria might exceed the recommended amounts, resulting in a higher rate of overweight/obesity. Other factors, such as the cooking methods might also influence the healthy properties of food. Analyzing in detail the dietary habits of our population sample, we found that the percentage of the schoolchildren population from Calabria declaring to go to a fast-food restaurant and consuming sweets and candy was statistically higher than the one from Lazio, which might explain the higher percentage of overweight/obese participants in Calabria. Indeed, a recent systematic review and meta-analysis of observational studies identified a higher intake of fast food and sugar-sweetened beverages as the primary dietary risk factors for overweight/obesity in children and adolescents [34]. In contrast, a higher consumption of FV in childhood is related to healthier outcomes in adulthood. In our study, a lower percentage of schoolchildren from Calabria declared to consume one fruit/day compared to the Lazio sample. This consumption is far from the WHO’s recommended intake, which should be 200 g of fruits and 300 g of vegetables distributed in about five portions per day, or at least 400 g/day of both FV [35]. It has been recently reported that 25.9% of Italian children consume FV less than once a day, and particularly 35.8% and 27.4% of children from Calabria and Lazio, respectively, had this unhealthy eating habit [36].

Moreover, the mean skin carotenoid score, which reflects FV intakes [37,38], measured by the Veggie Meter^®^, was below the optimal range, confirming data recently published in the same age range [21]. Similarly, we found no gender-related differences in the population sample from Calabria, while carotenoid levels were higher in boys than in girls from Lazio. Although the measurement of the skin carotenoid is a rapid and non-invasive procedure useful in screening studies, it measures the sum of carotenoids. Thus, it is not possible to assess the level of every single carotenoid, which requires the evaluation of carotenoid levels in the plasma sample, representing a limitation of this innovative device. It is important to note that we found significantly higher levels of skin carotenoids in schoolchildren from Lazio than in Calabria, with no differences when considering the frequency of FV intake, suggesting that geographical differences, such as the type and quality of FV consumed, might influence the carotenoid score. Moreover, the increased adipose tissue in the population from Calabria might affect the distribution and deposit of carotenoids in the skin. Indeed, it is considered one of the major storage organs for dietary carotenoids [39,40], thus influencing skin carotenoid levels. Data on the correlation between skin carotenoids and BMI in children are conflicting depending on the influence of different factors, including ethnicity, gender, and age range [41,42]. In our study, the carotenoid score was negatively associated with the BMI only in the schoolchildren from Lazio. Although further studies are needed to fully understand the relationship between skin carotenoids and anthropometric parameters, maintaining high carotenoid levels through FV consumption should be encouraged. Indeed, carotenoids, as potent natural antioxidants derived from diet, have beneficial effects on human health, exerting a protective role against a wide range of metabolic and chronic diseases, including diabetes mellitus, obesity, cardiovascular diseases, neurological disorders, and different types of cancer [43]. In particular, carotenoids can reduce oxidative stress and inflammatory reactions, enhancing the activity of antioxidant and cytoprotective phase II enzymes via the nuclear factor erythroid 2-related factor 2 (Nrf2) and the peroxisome proliferator-activated receptor (PPAR) [43]. Moreover, carotenoids have been shown to block adipocyte formation and decrease fat accumulation, preventing obesity. Thus, consumption of FV, enriched with natural antioxidant carotenoids, should be recommended to prevent the onset of several non-communicable diseases.

A limitation of this study is that children were recruited from primary schools and the results are not necessarily generalizable to other age groups or populations. Some of the variables were self-reported or reported by teachers or parents and may not have been completely objective. The use of BMI as the sole indicator of overweight and obesity may be a limitation because it does not distinguish between body fat and muscle mass. However, this is not crucial in epidemiological studies. Moreover, the body fat content of participants was not assessed. Finally, the type and portion size of FV consumed by the schoolchildren were not evaluated, making it difficult to make conclusions about the differences observed in the carotenoid scores of children from Calabria and Lazio.

## 5. Conclusions

This study confirms the anthropometric and eating habits differences between two schoolchildren populations from Central and Southern Italy, suggesting the need to promote a healthy lifestyle, including the consumption of FV as a dietary source of potent natural antioxidants to reduce obesity, especially in Southern Italy, as a strategy for the prevention of chronic NCDs over the entire lifespan. Further studies are essential to better understand the influence of anthropometric parameters on skin carotenoids and their potential role as indicators of health status in children.

## Figures and Tables

**Table 1:** Characteristics of the study population.

**Characteristics**	**Lazio**	**Calabria**	***p*-Value**
Subjects (n)	121	124	
Girls (n, %)	66, 54.54	66, 53.66	
Boys (n, %)	55, 45.45	58, 46.34	
Weight (kg)			
Total (Mean ± SD)	26.2 ± 5.92	36.24 ± 9.49	<0.0001
Girls (Mean ± SD)	26.06± 6.24	36.01 ± 9.05	<0.0001
Boys (Mean ± SD)	26.37 ± 5.55	36.5 ± 10.03	<0.0001
Height (cm)			
Total (Mean ± SD)	128.3 ± 9.06	136.9 ± 9.14	<0.0001
Girls (Mean ± SD)	127.9 ± 9.46	136.9 ± 9.13	<0.0001
Boys (Mean ± SD)	128.8 ± 8.62	136.9 ± 9.14	0.0004
BMI (kg/m^2^)			
Total (Mean ± SD)	15.77 ± 2.02	19.14 ± 3.61	<0.0001
Girls (Mean ± SD)	15.75 ± 2.03	19.13 ± 3.73	<0.0001
Boys (Mean ± SD)	15.79 ± 2.04	19.17 ± 3.51	<0.0001
BMI Z-score			
Total (Mean ± SD)	−0.23 ± 1.06	0.98 ± 1.26	<0.0001
Girls (Mean ± SD)	−0.21 ± 1.09	0.89 ± 1.37	<0.0001
Boys (Mean ± SD)	−0.24 ± 1.02	1.05 ± 1.16	<0.0001

BMI: body mass index; SD: standard deviation.

**Table 2:** Distribution of participants categorized by gender based on their BMI and BMI-Z score.

	Lazio	Calabria	*p*-Value
BMI			
Total			<0.0001
Underweight–normal weight (%)	88	48	
Overweight (%)	9	31	
Obese (%)	3	21	
Girls			<0.0001
Underweight–normal weight (%)	85	50	
Overweight (%)	12	31	
Obese (%)	3	19	
Boys			<0.0001
Underweight–normal weight (%)	91	46	
Overweight (%)	7	32	
Obese (%)	2	22	
BMI Z-score			
Total			<0.0001
Underweight–normal weight (%)	88	40	
Overweight (%)	11	30	
Obese (%)	1	30	
Girls			<0.0001
Underweight–normal weight (%)	86	42	
Overweight (%)	14	29	
Obese (%)	0	29	
Boys			<0.0001
Underweight–normal weight (%)	90	38	
Overweight (%)	8	31	
Obese (%)	2	31	

BMI: body mass index.

**Table 3:** Mean value of the adherence to the Mediterranean diet based on the KIDMED test.

	Total (Mean ± SD, n)	Girls (Mean ± SD, n)	Boys (Mean ± SD, n)	*p*-Value
Lazio	5.07 ± 2.25, 59	4.724 ± 2.186, 29	5.4 ± 2.298, 30	0.25
Calabria	5.47 ± 2.46, 121	5.515 ± 2.445, 65	5 ± 2.784, 56	0.27
*p*-Value	0.22	0.14	0.50	

SD: standard deviation.

**Table 4:** Compliance with items from the KIDMED test in the total sample. Frequency (%) of population distribution with respect to the cut-off points within or outside recommendations according to the KIDMED score.

Items	Lazio		Calabria		*p*-Value
	% Inside	% Outside	% Inside	% Outside	
Takes a fruit every day	90	10	80	20	0.047
Has a second fruit every day	53	47	58	42	
Has fresh or cooked vegetables regularly once per day	80	20	76	24	
Has fresh or cooked vegetables more than once per day	49	51	44	56	
Consumes fish regularly (at least 2–3 times per week)	58	42	49	51	
Likes pulses and eats them more than once per week	64	36	79	21	0.02
Consumes whole-grain pasta or whole-grain rice almost every day (5 or more times per week)	95	5	63	37	<0.0001
Has whole cereals or whole-grains (whole-meal bread, etc.) for breakfast	86	14	47	53	<0.0001
Consumes nuts regularly (at least 2–3 times per week)	27	73	48	52	0.003
Uses olive oil at home	98	2	89	11	0.003
Has a dairy product for breakfast (yoghurt, milk, etc.)	59	41	79	21	0.002
Takes two yogurts and/or some cheese (40 g) daily	22	78	64	36	<0.0001
Has commercially baked goods or pastries for breakfast	58	42	35	65	0.001
Goes to a fast-food (hamburger) restaurant more than once per week	20	80	67	33	<0.0001
Has breakfast	20	80	17	83	
Takes sweets and candy several times every day	29	71	53	47	<0.0006

**Table 5:** Mean carotenoid score measured by Veggie Meter^®^ in the total population and categorized by gender.

	Lazio (Mean ± SD)	Calabria (Mean ± SD)	*p*-Value
Total	315.3 ± 101.5	273.6 ± 83.03	0.0005
Girls	297.5 ± 94.2	269.3 ± 90.72	0.08
Boys	336.6 ± 106.6	278 ± 74.44	0.001
*p*-Value	0.03	0.56	

**Table 6:** Carotenoid score in the sample population based on the FV frequency intake.

	Lazio (Mean ± SD, n)	Calabria (Mean ± SD, n)	*p*-Value
Item #1			
<2 servings/day	266.1 ± 110.3, 28	252.7 ± 62.48, 51	0.49
>2 servings/day	323.8 ± 75.14, 31	289.2 ± 93.39, 69	0.07
*p*-Value	0.02	0.02	
Item #2			
<2 servings/day	297 ± 116.5, 31	268.8 ± 84.3, 69	0.17
>2 servings/day	299.6 ± 73.53, 29	280.4 ± 82.47, 51	0.30
*p*-Value	0.92	0.45	

**Table 7:** Multiple regression models between carotenoid score and different parameters in the entire sample population.

	β (95% CI)	SE	*p*-Value
Gender [F]	−23.15 (−48.56; −2.27)	12.88	0.07
BMI	−0.73 (−4.78; 3.33)	2.05	0.72
>2 servings of fruit/day (>2 = 1)	41.25 (14.56; 67.94)	13.52	0.003
>2 servings of vegetable/day (>2 = 1)	−1.62 (−27.55; 24.32)	13.14	0.90
Italian Region (Lazio = 1)	23.19 (−6.66; 53.04)	15.12	0.13
Adj.R^2^	0.07		

## Data Availability

The data presented in this study are available in this article.

## Abbreviations

The following abbreviations are used in this manuscript:

BMI	Body Mass Index
FV	Fruits and Vegetables
MD	Mediterranean Diet
NCDs	Non-Communicable Diseases

## References

[1] https://www.who.int/europe/initiatives/who-european-childhood-obesity-surveillance-initiative-(cosi).

[2] https://www.epicentro.iss.it/okkioallasalute/.

[3] Roblin L. (2007). Childhood obesity: Food, nutrient, and eating-habit trends and influences. Appl. Physiol. Nutr. Metab..

[4] Kuzbicka K., Rachon D. (2013). Bad eating habits as the main cause of obesity among children. Pediatr. Endocrinol. Diabetes Metab..

[5] Sahoo K., Sahoo B., Choudhury A.K., Sofi N.Y., Kumar R., Bhadoria A.S. (2015). Childhood obesity: Causes and consequences. J. Fam. Med. Prim. Care.

[6] Boeing H., Bechthold A., Bub A., Ellinger S., Haller D., Kroke A., Leschik-Bonnet E., Muller M.J., Oberritter H., Schulze M. (2012). Critical review: Vegetables and fruit in the prevention of chronic diseases. Eur. J. Nutr..

[7] Ruel G., Shi Z., Zhen S., Zuo H., Kroger E., Sirois C., Levesque J.F., Taylor A.W. (2014). Association between nutrition and the evolution of multimorbidity: The importance of fruits and vegetables and whole grain products. Clin. Nutr..

[8] Wallace T.C., Bailey R.L., Blumberg J.B., Burton-Freeman B., Chen C.O., Crowe-White K.M., Drewnowski A., Hooshmand S., Johnson E., Lewis R. (2020). Fruits, vegetables, and health: A comprehensive narrative, umbrella review of the science and recommendations for enhanced public policy to improve intake. Crit. Rev. Food Sci. Nutr..

[9] Folkvord F., Naderer B., Coates A., Boyland E. (2021). Promoting Fruit and Vegetable Consumption for Childhood Obesity Prevention. Nutrients.

[10] Smith L., Lopez Sanchez G.F., Veronese N., Soysal P., Oh H., Barnett Y., Keyes H., Butler L., Allen P., Kostev K. (2022). Fruit and Vegetable Intake and Non-Communicable Diseases among Adults Aged >/=50 Years in Low- and Middle-Income Countries. J. Nutr. Health Aging.

[11] Library of Evidence for Nutrition Actions (eLENA). https://www.who.int/tools/elena/interventions/.

[12] Slavin J.L., Lloyd B. (2012). Health benefits of fruits and vegetables. Adv. Nutr..

[13] Marti R., Rosello S., Cebolla-Cornejo J. (2016). Tomato as a Source of Carotenoids and Polyphenols Targeted to Cancer Prevention. Cancers.

[14] Koklesova L., Liskova A., Samec M., Buhrmann C., Samuel S.M., Varghese E., Ashrafizadeh M., Najafi M., Shakibaei M., Busselberg D. (2020). Carotenoids in Cancer Apoptosis-The Road from Bench to Bedside and Back. Cancers.

[15] Di Mascio P., Kaiser S., Sies H. (1989). Lycopene as the most efficient biological carotenoid singlet oxygen quencher. Arch. Biochem. Biophys..

[16] Stahl W., Sies H. (2003). Antioxidant activity of carotenoids. Mol. Asp. Med..

[17] Harrison E.H. (2012). Mechanisms involved in the intestinal absorption of dietary vitamin A and provitamin A carotenoids. Biochim. Biophys. Acta.

[18] Rodriguez-Concepcion M., Avalos J., Bonet M.L., Boronat A., Gomez-Gomez L., Hornero-Mendez D., Limon M.C., Meléndez-Martínez A.J., Olmedilla-Alonso B., Palou A. (2018). A global perspective on carotenoids: Metabolism, biotechnology, and benefits for nutrition and health. Prog. Lipid Res..

[19] Meléndez-Martínez A.J. (2019). An Overview of Carotenoids, Apocarotenoids, and Vitamin A in Agro-Food, Nutrition, Health, and Disease. Mol. Nutr. Food Res..

[20] Longevity Link Corporation The Veggie Meter^®^: Non-Invasive Optical Biomarker for Personal Health. http://www.longevitylinkcorporation.com.

[21] Caparello G., Ceraudo F., Meringolo F., Augimeri G., Morino G., Bonofiglio D. (2024). Eating habits and carotenoid skin content among children based on their attendance at the school meals: A cross-sectional pilot study. J. Clin. Transl. Endocrinol..

[22] Morelli C., Avolio E., Galluccio A., Caparello G., Manes E., Ferraro S., De Rose D., Santoro M., Barone I., Catalano S. (2020). Impact of Vigorous-Intensity Physical Activity on Body Composition Parameters, Lipid Profile Markers, and Irisin Levels in Adolescents: A Cross-Sectional Study. Nutrients.

[23] Serra-Majem L., Ribas L., Ngo J., Ortega R.M., Garcia A., Perez-Rodrigo C., Aranceta J. (2004). Food, youth and the Mediterranean diet in Spain. Development of KIDMED, Mediterranean Diet Quality Index in children and adolescents. Public Health Nutr..

[24] Kim J., Lim H. (2019). Nutritional Management in Childhood Obesity. J. Obes. Metab. Syndr..

[25] Liberali R., Kupek E., Assis M.A.A. (2020). Dietary Patterns and Childhood Obesity Risk: A Systematic Review. Child. Obes..

[26] Lassale C., Fito M., Morales-Suarez-Varela M., Moya A., Gomez S.F., Schroder H. (2022). Mediterranean diet and adiposity in children and adolescents: A systematic review. Obes. Rev..

[27] Pereira A.R., Oliveira A. (2021). Dietary Interventions to Prevent Childhood Obesity: A Literature Review. Nutrients.

[28] Acito M., Valentino R., Rondini T., Fatigoni C., Moretti M., Villarini M. (2024). Mediterranean Diet Adherence in Italian Children: How much do Demographic Factors and Socio-Economic Status Matter?. Matern. Child Health J..

[29] Cardamone E., Di Benedetto R., Lorenzoni G., Gallipoli S., Ghidina M., Zobec F., Iacoponi F., Gregori D., Silano M. (2023). Adherence to Mediterranean diet in Italy (ARIANNA) cross-sectional survey: Study protocol. BMJ Open.

[30] Garcia-Conesa M.T., Philippou E., Pafilas C., Massaro M., Quarta S., Andrade V., Jorge R., Chervenkov M., Ivanova T., Dimitrova D. (2020). Exploring the Validity of the 14-Item Mediterranean Diet Adherence Screener (MEDAS): A Cross-National Study in Seven European Countries around the Mediterranean Region. Nutrients.

[31] Caparello G., Galluccio A., Giordano C., Lofaro D., Barone I., Morelli C., Sisci D., Catalano S., Ando S., Bonofiglio D. (2020). Adherence to the Mediterranean diet pattern among university staff: A cross-sectional web-based epidemiological study in Southern Italy. Int. J. Food Sci. Nutr..

[32] Augimeri G., Soto M., Ceraudo F., Caparello G., Villegas Figueroa M., Cesario M., Caputi L.S., Calderon B., Bonofiglio D. (2024). Differences of skin carotenoids and adherence to the Mediterranean Diet pattern in adults from Southern Italy and Dominican Republic. J. Transl. Med..

[33] Nardone P., Ciardullo S., Mandolini D., Salvatore M.A., Spinelli A. Lo Stato Ponderale dei Bambini e delle Bambine OKkio alla SALUTE: Rilevazione Dati 2023. Centro Nazionale di Prevenzione delle Malattie e Promozione della Salute, Istituto Superiore di Sanità, 10 May 2024 Roma, Italy. https://www.epicentro.iss.it/okkioallasalute/pdf2024/convegno-2024/Conv.10-Mag%2010.40%20-%20Lo%20stato%20ponderale%20dei%20bambini%20e%20delle%20bambine%20-%20Nardone%20Ciardullo.pdf.

[34] Jakobsen D.D., Brader L., Bruun J.M. (2023). Association between Food, Beverages and Overweight/Obesity in Children and Adolescents-A Systematic Review and Meta-Analysis of Observational Studies. Nutrients.

[35] World Health Organization Increasing Fruit and Vegetable Consumption to Reduce the Risk of Noncommunicable Diseases. https://www.who.int/tools/elena/interventions/fruit-vegetables-ncds.

[36] Spinelli A., Mandolini D., Salvatore M.A. (2024). Gli stili di vita dei bambini e delle bambine. Proceedings of the Stato Ponderale e Stili di Vita di Bambine e Bambini: I Dati Italiani della Sorveglianza “OKkio alla SALUTE 2023” e il Contributo dello Studio EPaS-ISS.

[37] Martinelli S., Acciai F., Tasevska N., Ohri-Vachaspati P. (2021). Using the Veggie Meter in Elementary Schools to Objectively Measure Fruit and Vegetable Intake: A Pilot Study. Methods Protoc..

[38] Augimeri G., Soto M., Ceraudo F., Caparello G., Villegas Figueroa M., Cesario M., Caputi L.S., Calderon B., Bonofiglio D. (2024). Comparing the Dietary Habits and the Food Choices Between Italian and Dominican Adult Populations: Focus on Fruit and Vegetable Intakes and Their Association with Skin Carotenoid Levels. Foods.

[39] Parker R.S. (1989). Carotenoids in Human Blood and Tissues. J. Nutr..

[40] Kaplan L.A., Lau J.M., Stein E.A. (1990). Carotenoid composition, concentrations, and relationships in various human organs. Clin. Physiol. Biochem..

[41] Madore M.P., Hwang J.E., Park J.Y., Ahn S., Joung H., Chun O.K. (2023). A Narrative Review of Factors Associated with Skin Carotenoid Levels. Nutrients.

[42] Seguin-Fowler R.A., Hanson K.L., Marshall G.A., Belarmino E.H., Jilcott Pitts S.B., Kolodinsky J., Sitaker M., Ammerman A. (2021). Fruit and Vegetable Intake Assessed by Repeat 24 h Recalls, but Not by A Dietary Screener, Is Associated with Skin Carotenoid Measurements in Children. Nutrients.

[43] Fiedor J., Burda K. (2014). Potential role of carotenoids as antioxidants in human health and disease. Nutrients.

